# Combined ATR and DNA-PK Inhibition Radiosensitizes Tumor Cells Independently of Their p53 Status

**DOI:** 10.3389/fonc.2018.00245

**Published:** 2018-07-13

**Authors:** Hind Hafsi, Magnus T. Dillon, Holly E. Barker, Joan N. Kyula, Ulrike Schick, James T. Paget, Henry G. Smith, Malin Pedersen, Martin McLaughlin, Kevin J. Harrington

**Affiliations:** ^1^Division of Radiotherapy and Imaging, The Institute of Cancer Research, London, United Kingdom; ^2^The Royal Marsden Hospital, London, United Kingdom; ^3^Radiation Oncology Department, University Hospital Morvan, Brest, France

**Keywords:** DNA damage response, radiosensitization, checkpoint abrogation, mitotic catastrophe, ataxia telangiectasia- and RAD3-related

## Abstract

Head and neck squamous cell carcinoma (HNSCC) is a significant cause of cancer deaths. Cisplatin-based chemoradiotherapy is a standard of care for locally advanced disease. ATR and DNA-PK inhibition (DNA-PKi) are actively being investigated in clinical trials with preclinical data supporting clinical translation as radiosensitizers. Here, we hypothesized that targeting both ATR and DNA-PK with small molecule inhibitors would increase radiosensitization of HNSCC cell lines. Radiosensitization was assessed by Bliss independence analysis of colony survival data. Strong cell cycle perturbing effects were observed with ATR inhibition reversing the G2/M arrest observed for radiation-DNA-PKi. Increased apoptosis in combination groups was measured by Sub-G1 DNA populations. DNA-PKi increased radiation-induced RAD51 and gamma-H2Ax foci, with the addition of ATR inhibition reducing levels of both. A sharp increase in nuclear fragmentation after aberrant mitotic transit appears to be the main driver of decreased survival due to irradiation and dual ATR/DNA-PKi. Dual inhibition of DNA-PK and ATR represents a novel approach in combination with radiation, with efficacy appearing to be independent of p53 status. Due to toxicity concerns, careful assessment is necessary in any future translation of single or dual radiosensitization approaches. Ongoing clinical trials into the ATR inhibitor AZD6738 plus radiation, and the phenotypically similar combination of AZD6738 and the PARP inhibitor olaparib, are likely to be key in ascertaining the toxicity profile of such combinations.

## Introduction

In human cells, non-homologous end-joining (NHEJ) and homologous recombination (HR) are the major DNA double-strand breaks (DSBs) repair pathways, and defects in these pathways cause genome instability and promote tumorigenesis. DSBs can be formed (i) following exposure to ionizing radiation (X- or γ-rays) or genotoxic drugs, (ii) endogenously, during DNA replication, or (iii) as a consequence of reactive oxygen species generated during oxidative metabolism.

Radiotherapy is a standard of care for head and neck squamous cell carcinoma (HNSCC). The development of intensity-modulated radiotherapy has helped to spare normal tissues, such as the parotid gland, from radiation-induced toxicity by more accurately targeting head and neck tumors (1). While novel techniques, such as the image-guided MR-Linac, are entering trials (2), there are physical limits to significant further escalation of radiation doses due to normal tissue toxicity. Tumor-specific radiosensitizers targeting DNA damage response (DDR) pathways represent a novel means to enhance the efficacy of radiotherapy.

The cell cycle phase in which the DSB occurs will determine the choice of the repair mechanism used by the cell (3, 4). NHEJ links the two ends of broken DNA by direct ligation and is active during all phases of the cell cycle, particularly during G0, G1, and early S phase. HR is most predominant during late S and G2 phases when a sister chromatid can be used as a template for repair.

The PI3-kinase like family of protein kinases includes ATM, ATR, and DNA-PKcs, which recruit DNA repair proteins and activate cell cycle checkpoints in response to DSBs (5). Most cancer cells are defective in the G1 checkpoint and, therefore, rely on their S/G2 checkpoints for survival following DNA damage. This may represent a means to specifically radiosensitize tumor cells. Of specific relevance to HNSCC is the loss of function of p53 by mutation, or degradation by E6 produced as a result of infection with high-risk human papillomavirus genotypes such as HPV16 (6, 7).

ATR, which initiates the S and G2 cell cycle checkpoint cascade by phosphorylating CHK1, is an attractive target in cancer therapy. ATR acts as a key kinase in DDR signaling. DNA lesions which result in single-stranded DNA are coated by the protective replication protein A. ATR subsequently binds *via* the partner protein ATRIP. ATR acts both to stabilize stalled replication forks and to initiate downstream signaling *via* CHK1 leading to cell cycle arrest. A number of preclinical reports have demonstrated sensitization by ATR inhibitors to chemotherapy and radiotherapy (8–12).

Two ATR inhibitors, such as VX-970 and AZD6738, are currently undergoing clinical evaluation. We are currently assessing safety of AZD6738 in a phase I clinical trial (PATRIOT), alone and in combination with radiotherapy (NCT02223923). Other trials are currently evaluating AZD6738 in combination with paclitaxel, carboplatin, olaparib, or durvalumab (NCT02630199, NCT02264678).

DNA-PK, a key component of the NHEJ pathway, correlates with decreased response to DNA-damaging agents and therapeutic resistance in multiple cancers (13–15). Furthermore, DNA-PK has also been implicated in other tumor-associated processes, including genomic stability, hypoxia, inflammatory response, and transcription (16). Thus, the inhibition of DNA-PK is an attractive approach selectively to enhance DNA-damaging therapies. As such, DNA-PK has received significant attention from both industry and academia as a potential anticancer target. Many of the first identified DNA-PK inhibitors suffered from lack of specificity and poor pharmacokinetic properties, but more recently developed inhibitors, including NU7441 and KU-0060648, demonstrate improved specificity and pharmaceutical properties (17) and have been used preclinically to explore the impact of DNA-PK inhibition (DNA-PKi) in multiple tumor types (18–20). In the clinic, two DNA-PK targeting molecules, such as CC-122 and CC-115, are currently being evaluated. CC-122 is in a phase I clinical trial (NCT01421524) for solid tumors, non-Hodgkin lymphoma, and multiple myeloma. CC-115 is a dual inhibitor of DNA-PK and mTOR and is currently in phase I trial (NCT01353625) for advanced solid tumors and hematologic malignancies.

Considering that dysfunction of one DDR pathway may be compensated by another repair pathway (21) and contribute to resistance to DNA-damaging radiation and chemotherapies, we hypothesized that targeting both NHEJ and HR with small molecule inhibitors would enhance the radiosensitivity of tumor cells. Canonically, ATR is active during DNA replication in S phase, while repair by DNA-PK is utilized during non-replicative cell cycle phases such as G1. Inhibition of both targets simultaneously may overcome resistance in phases of the cell cycle where activity of each single target is lower or absent. We have previously shown that radiosensitization by AZD6738 is dependent on proliferation (8). We, therefore, used the ATR inhibitor, AZD6738, and the DNA-PK inhibitor, KU-0060648, and sought to identify favorable interactions between them. Due to the absence of available p53 isogenic models for HNSCC, we used the isogenic HCT116 p53+/+ and HCT116 p53−/− model to more conclusively study the role of p53 loss.

We demonstrate that AZD6738 and KU-0060648 both potentiate the sensitivity to radiation as single agents and appear to be at least additive when combined together. Mechanistic analyses *in vitro* indicated reduced survival and a combined perturbation of cell cycle kinetics and DNA repair, resulting in severe nuclear fragmentation.

## Materials and Methods

### Cell Culture Conditions

LICR-LON-HN4 and LICR-LON-HN5 were a kind gift from Suzanne Eccles (The Institute of Cancer Research, Sutton, London, UK). HCT116 p53+/+ and p53−/− cells were obtained from Horizon Discovery (Cambridge, UK). Cells were cultured in DMEM (Invitrogen, Paisley, UK) supplemented with 10% FCS, 2 mM l-glutamine, and 1% penicillin/streptomycin at 37°C with 5% CO_2_. Under these conditions, HCT116 and HN5 cell lines displayed doubling times of 20–24 h, HN4 30–36 h. Cells were tested for *Mycoplasma* using the eMyco PCR kit from IntroBio (Seongnam-Si, South Korea) and authenticated by STR profiling (Bio-Synthesis Inc., TX, USA).

### Drugs and Irradiation

AZD6738 and KU-0060648 were kindly donated by AstraZeneca. Irradiation was carried out using an AGO 250 kV X-ray machine (AGO, Reading, UK).

### Survival Assays

For MTT assays, cells were plated in 96-well plates and, after 24 h, treated with AZD6738 and KU-0060648. Cell survival was measured 48 h after treatment by 3-(4,5-dimethylthiazol-2-yl)-2,5-diphenyltetrazolium bromide (MTT) assay according to the manufacturer’s instructions. Long-term survival in response to radiation was measured by colony formation assay. Cells were seeded in 6-well dishes at appropriate densities. Cells were allowed to attach overnight. AZD6738 and KU-0060648 were added at the indicated concentrations 1 h before irradiation, drugs were not removed post-radiation. After 14 days, colonies were fixed and stained in 5% glutaraldehyde, 0.5% crystal violet, with colonies containing more than 50 cells counted.

### Western Blotting

Cells were plated in 10-cm dishes and allowed to attach overnight. AZD6738 and KU-0060648 were added at the indicated concentrations 1 h before irradiation. Medium and cells were harvested in PBS-containing 1 mM Na_3_VO_4_ and 1 mM NaF. Cells were pelleted before lysis in 50 mM Tris–HCl pH 7.5, 150 mM NaCl, 1% NP-40, 0.5% deoxycholate, and 0.1% SDS. Lysis supernatants were quantified by BCA assay from Pierce (Leicestershire, UK). Total protein lysate was separated by reducing SDS-PAGE, transferred to Midi Nitrocellulose membranes using a Transblot Turbo (Bio-Rad, Watford, UK), and blocked with TBS blocking buffer (Li-Cor Biotechnology, Cambridge, UK). Membranes were probed with antibodies specific for GAPDH clone 10C10, CHK1 clone 2G1D5, phospho-CHK1 (S345) clone 133D3, DNA-PK code 4602, γH2Ax (S139) clone 20E3 and Caspase 3 clone 8G10 from Cell Signaling (MA, USA); phospho-DNA-PK from Abcam ab18192 (Cambridge, UK); PARP clone F-2 was purchased from Santa Cruz (TX, USA).

### Cell Cycle

Cells were plated in 10-cm dishes and allowed to attach overnight. AZD6738 and KU-0060648 were added at the indicated concentrations 1 h before irradiation. Medium and cells were harvested at the desired time point after treatment and fixed overnight in ethanol. Cells were subsequently rehydrated in PBS and stained with propidium iodide and RNase solution from Cell Signaling (MA, USA). Cells were stained for mitotic index using anti-phospho-histone H3 (S10) AlexaFluor 647 from Cell Signaling (MA, USA). BrdU staining was performed by incubating cells with BrdU before collection and fixation in ethanol. Cells were subsequently stained using anti-BrdU antibody from Dako/Agilent (Stockport, UK). Cells were analyzed on an LSR II from BD Biosciences (Oxford, UK).

### Confocal Microscopy

Cells were plated in 35-mm glass-bottomed dishes from Mattek (MA, USA) and allowed to attach overnight. AZD6738 and KU-0060648 were added 1 h before irradiation. Samples were fixed in 10% neutral buffered formalin, permeabilized in 0.2% Triton X-100, and treated with DNaseI from Roche (West Sussex, UK). Cells were blocked in 1% BSA, 2% FCS in PBS before staining using antibodies for γH2Ax (S139) clone 20E3 from Cell Signaling (MA, USA), or RAD51 clone 14B4 from Genetex (CA, USA) with goat anti-rabbit AlexaFluor 488 or goat anti-mouse AlexaFluor 546 as secondary antibodies from Invitrogen (Paisley, UK). Nuclei were counterstained with DAPI. Samples were imaged using a Zeiss LSM710 inverted confocal microscope (Jena, Germany). Individual nuclei were scored manually for normal morphology, nuclear fragmentation such as micronuclei, or gross lobular irregularities. Nuclei were also scored for γH2Ax foci. >5 γH2Ax were scored as positive. Pan-nuclear γH2Ax staining was also scored if γH2Ax foci numbers were unquantifiable. RAD51 and γH2Ax foci were also quantified by automated image quantification using Cell Profiler v2.2 (Broad Institute, CA, USA). Nuclei were segmented and counted based on DAPI staining, RAD51 and γH2Ax were counted and expressed as average foci per nucleus. Quantification of each independent experiment included approximately 100–300 nuclei.

### Statistical Analysis

**Figure 1:**
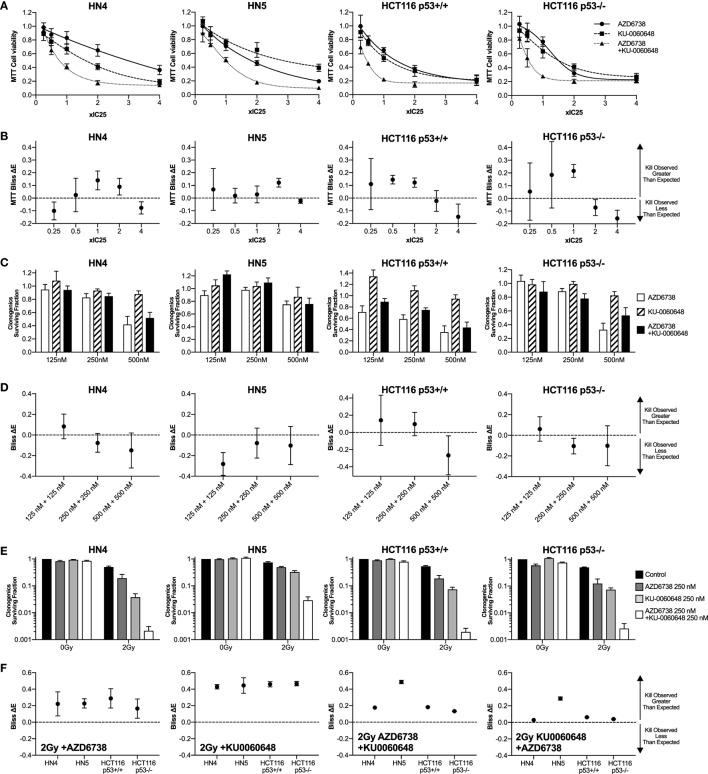

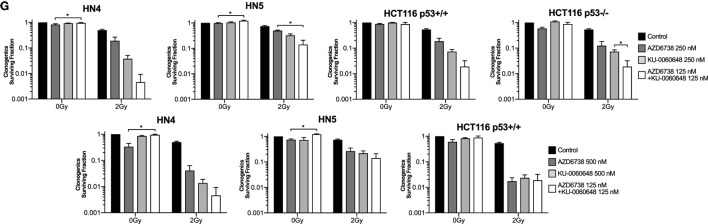
DNA-PK inhibition enhances radiosensitization when added to ATR inhibition and radiation. **(A)** The combination of the ATR inhibitor AZD6738 and the DNA-PK inhibitor KU-0060648 was assessed by first determining cell viability 48 h after treatment by MTT analysis at 0.25×, 0.5×, 1×, 2×, and 4× IC_50_ doses. Dose–response curves were used initially to derive the following IC_50_ doses. For AZD6738, HN4, 1.5 µM; HN5, 1.5 µM; HCT116 p53+/+, 3.0 µM; and HCT116 p53−/−, 4.0 µM. For KU-0060648, HN4, 0.8 µM; HN5, 0.5 µM; HCT116 p53+/+, 1.5 µM; and HCT116 p53−/−, 1.5 µM. In subsequent combination experiments, these doses proved to be approximately IC_25_ doses and have been presented accordingly for accuracy. **(B)** Observed cell kill vs expected cell kill was determined by Bliss independence analysis. For example, ×1 IC_50_ + ×1 IC_50_ is the observed toxicity of this combination (expressed as 0 to 1 with 1 being maximum kill), minus the calculated “expected” effect if the individual values for ×1 IC_50_ AZD6738 and ×1 IC_50_ KU0060648 were expected to combine without any interaction. A positive Δ*E* corresponds to greater observed kill than expected for non-interacting combinations. Negative Δ*E* indicates less than expected cell kill. Further details outlined in the statistical analysis section of the methods. Greater than expected cell kill is indicated by Δ*E* and 95% confidence intervals (CIs) from observed data falling all above zero; less than expected cell kill where all values fall below zero. **(C)** Clonogenic survival was determined for AZD6738 and KU-0060648 alone and in combination across a range of doses. Values expressed as surviving fraction relative to untreated control. **(D)** Bliss analysis as described in panel **(B)**. **(E)** Clonogenic survival was determined for combinations of AZD6738, KU-0060648, and 2 Gy radiation. Values expressed as surviving fraction relative to untreated control. **(F)** Bliss analysis as described in panel **(B)**. **(G)** Comparison plots of the combination of 125 nM each of AZD6738 and KU0060648 vs 250 or 500 nM of individual drugs and the corresponding radiosensitization effect by clonogenics. All panels represent a minimum of three independent experiments ±SEM, except Bliss analysis in **(B,D,F)** where error bars are ±95% CIs. Statistical analysis performed between indicated conditions by unpaired *t*-test *P < 0.05.

Drug interaction in Figure 1 was assessed by Bliss independence analysis using the equation *E*_exp_ = *E*_x_ + *E*_y_ − (*E*_x_*E*_y_) (22). *E*, in this case, represents cell kill from 0 to 1 with 1 being maximum cell kill. *E*_exp_ is the expected effect if two treatments are additive with *E*_x_ and *E*_y_ corresponding to the effect of each treatment individually. Δ*E* = *E*_observed_ − *E*_exp_. Differences in observed vs expected cell kill is represented by Δ*E* ± 95% confidence intervals from observed data. Greater than expected cell kill is indicated by positive Δ*E* values, less than expected cell kill by negative Δ*E* values. In all other figures, unpaired two-tailed Student’s *t*-test was utilized for parametric analysis using Graphpad prism (version 7c).

## Results

### AZD6738 and KU-0060648 Combination Results in Improved Radiosensitization

A 4 cell line panel was initially selected with two p53 mutant (p53mt) HNSCC cell lines, HN4 and HN5, tested alongside the p53+/+ and p53−/− isogenic HCT116 cell lines. The p53 proficiency/deficiency of isogenic HCT116 cell lines has previously been validated in our lab by measuring p21 induction due to radiation (8). We first assessed cytotoxicity of AZD6738 and KU-0060648 alone or in combination by MTT over a range of doses (Figure 1A). IC_50_ doses of both AZD6738 and KU-0060648 were broadly similar across the 4 cell line panel, ranging from 1.5 to 4 and 0.5 to 1.5 µM, respectively. IC_50_ values used for combination MTT experiments in Figure 1A were found to be closer to IC_25_ and have been plotted as such. Bliss independence analysis was performed to determine if greater or less cell kill was observed vs calculated expected values when comparing different conditions. Bliss independence modeling indicated greater than expected cell kill to some degree at 0.5× or 1× IC_25_ values in a number of the cell lines, but less at other doses (Figure 1B). While both drugs are cytotoxic, rather than cytostatic, at higher concentrations and longer durations of exposure, the drop at these higher doses may be in part due to dynamic range limitations of the MTT assay at the time point used.

The combination of AZD6738 and KU-0060648 was then assessed by clonogenic assay across a range of doses (Figure 1C) with Bliss independence analysis performed as per MTT data previously. These results showed no clear trend for greater or less observed cell kill between AZD6738 and KU-0060648 when measured by clonogenic assay.

The ability of AZD6738 and KU-0060648 to radiosensitize alone, or as a dual radiosensitizer drug combination vs single radiosensitizer combinations was assessed by clonogenic assay (Figure 1E) and Bliss independence analysis (Figure 1F). Bliss analysis indicated that both AZD6738 and KU-0060648 alone acted as strong radiosensitizers in combination with radiation in all four cell lines. Greater than expected cell kill was observed in all cell lines for both the addition of AZD6738 to KU-0060648-radiation and the addition of KU-0060648 to AZD6738-radiation. Similar results were observed across a range of drug doses (complete data shown in Figure S1 in Supplementary Material). For 125 + 125 nM of each drug (250 nM combined) the radiosensitization effect observed is of equal potency to that observed for 500 nM of each drug alone (Figure 1G). This may present an opportunity for a reduced combined drug dose clinically. Combination of both drugs at 125 nM in the absence of radiation appears to have a lower toxicity vs single 500 nM doses, though this was only statistically significant for AZD6738. Combining both drugs at 125 nM had a stronger radiosensitization effect than either drug individually at 250 nM. These data are suggestive of potentially greater than additive effects.

**Figure 2:**
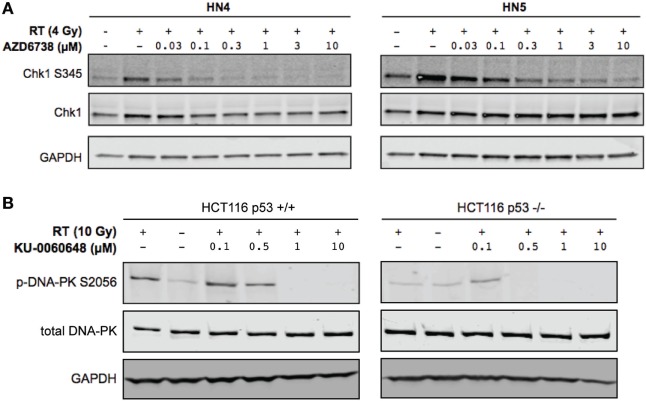
DNA-PK inhibition and ATR inhibition modulate DNA damage response signaling. **(A)** Western blot indicates AZD6738 inhibition of ATR through loss of downstream phosphorylation of CHK1 on Ser345. Dose escalation of AZD6738 with 4 Gy radiation, samples collected 1 h post-radiation. **(B)** Modulation of DNA-PK phosphorylation by KU-0060648 was shown by Western blot in HCT116 p53+/+ and p53−/− cell lines. Dose escalation of KU-0060648 with 10 Gy radiation, samples collected 1 h post-radiation.

Clonogenic assays may better represent the effect of DDR inhibitors alone or in combination with radiation when compared with MTT assay due to their long-term nature. While short-term MTT observations will be dominated by cell cycle-linked effects, such as death due to replicative stress or mitotic catastrophe, long-term clonogenic assays will also include toxicity as a consequence of misrepair of DNA damage. The results observed give a rationale for testing AZD6738 and KU-0060648 as a dual radiosensitizer treatment. Drug-on-target modulation of CHK1 on Ser345 and DNA-PK on Ser2056 was observed by Western blot (Figure 2). A clear reduction in radiation-induced Ser345 CHK1 downstream of ATR was observed starting at 30–100 nM AZD6738 concentrations. Ser2056 is an autophosphorylation site of DNA-PKcs which has previously been shown to be reduced by DNA-PKcs kinase inhibition (23). Clear inhibition was observed at 1 h post-radiation at concentrations greater than 100 nM.

### AZD6738 and KU-0060648 Modulate Cell Cycle Progression After Radiation

**Figure 3:**
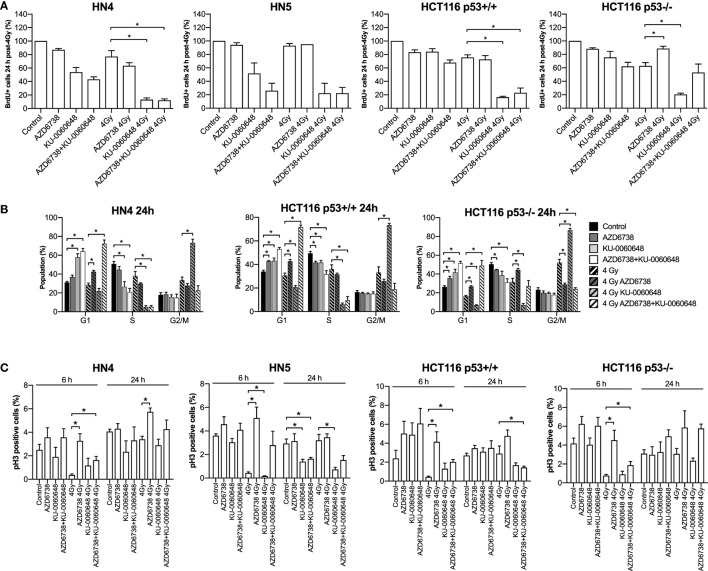
Modulation of the cell cycle by DNA-PK inhibition and ATR inhibition in combination with radiation. **(A)** FACS analysis of BrdU incorporation was used to assess active DNA synthesis in response to AZD6738, KU-0060648, and 4 Gy radiation combinations at 24 h post-radiation. Values expressed relative to control. **(B)** Cell cycle profiles at 24 h were assessed by staining for DNA content using propidium iodide in response to AZD6738, KU-0060648, and 4 Gy radiation combinations. **(C)** Cells in mitosis positive for phospho-histone H3 were quantified by FACS. Quantification was performed at 6 and 24 h in response to AZD6738, KU-0060648, and 4 Gy radiation combinations. All panels represent a minimum of three independent experiments ± SEM, except HN5 in **(A)** which is *n* = 2. Statistical analysis performed between indicated conditions by unpaired *t*-test *P < 0.05.

We further evaluated the impact of ATR, DNA-PKi, and irradiation on cell cycle distribution. Active DNA synthesis was measured by BrdU incorporation (Figure 3A). Variable reduction in BrdU incorporation was seen in all cell lines due to AZD6738, KU-0060648, or radiation monotherapy treatments at 24 h. The most significant reduction observed was due to the combination of radiation and KU-0060648. AZD6738 addition to radiation alone or radiation-KU-0060648 combined, did not appear to alter BrdU incorporation apart from a small increase observed in HCT116 p53+/+ (Figure 3A).

Cell cycle analysis by propidium iodide (Figure 3B) or measurement of the mitotic index by phospho-HistoneH3 (Figure 3C) was also performed. At 24 h, compared to radiation, AZD6738-radiation, or KU-0060648-radiation, the triple combination of AZD6738, KU-0060648, and radiation resulted in a significant increase in cells in G1 at the expense of S/G2. This was particularly marked for the addition of AZD6738 to KU-0060648-radiation. KU-0060648 induced a significant G2/M population increase when added to radiation, which was abrogated by the addition of AZD6738. Phospho-histoneH3 (Figure 3C) data at 6 h did not indicate any significant modulation of the mitotic index by KU-0060648, while AZD6738 abrogated any radiation-induced decrease in mitotic index. A lower mitotic index was observed in HN5 cell lines at 24 h in conditions containing KU-0060648. However, no other clear patterns could be identified except for an elevated mitotic index across conditions containing AZD6738.

### The Combination of AZD6738 and KU-0060648 With Radiation Increases the Sub-G1 Population

**Figure 4:**
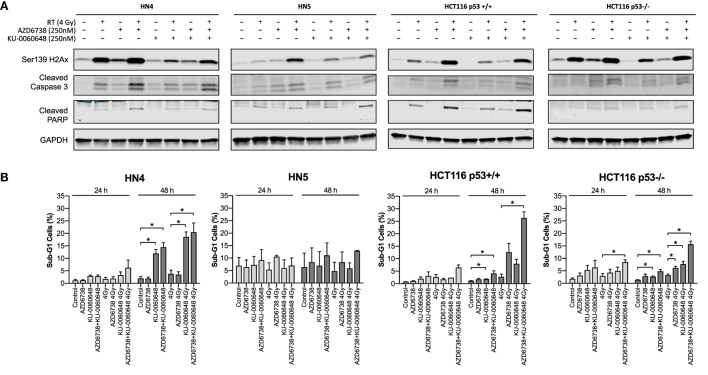
Apoptotic signaling in response to DNA-PK inhibition and ATR inhibition in combination with radiation. **(A)** Western blot analysis was used to assess levels of H2Ax phosphorylation on Ser139, and apoptosis was identified by cleaved caspase 3 and cleaved PARP. Cell lysates were harvested at 48 h after radiation with AZD6738 and KU-0060648 addition 1 h before radiation. **(B)** Sub-G1 cells were identified at 24 and 48 h by propidium iodide staining and FACS in response to AZD6738, KU-0060648, and 4 Gy radiation combinations. Data represent a minimum of three independent experiments ± SEM. Statistical analysis performed between indicated conditions by unpaired *t*-test *P < 0.05.

We further evaluated whether reduced survival and alterations to cell cycle progression corresponded to apoptotic cell death by measuring caspase-3, PARP cleavage, and γH2Ax levels at 48 h post-treatment (Figure 4A). AZD6738 and KU-0060648 as single agents did not, or only slightly, induced caspase-3 or PARP cleavage in all cells. The combination of AZD6738 and radiation induced a substantial increase in cleaved caspase-3 or cleaved PARP across all cell lines. Interestingly, KU-0060648 plus radiation, despite profound radiosensitization in clonogenic assays, did not result in increased PARP and caspase-3 cleavage. The addition of KU-0060648 resulted in no increase, or in some cases a decrease, in the cleavage observed when added to AZD6738-radiation. The addition of KU-0060648 did result in a clear decrease in γH2Ax signal in all cell lines tested (Figure 4A). This has previously been shown to indicate DNA-PKcs kinase inhibition (24) and as a clear sign of drug-on-target effect at the 250 nM dose used.

Analysis of DNA fragmentation and sub-G1 populations was carried out by propidium iodide cell cycle analysis (Figure 4B). This did reveal an increase in the sub-G1 population due to the addition of KU-0060648 to AZD6738-radiation which was particularly clear for HCT116 p53+/+ and p53−/− cell lines.

### AZD6738 Reduces RAD51 and γH2AX Foci Formation Induced by KU-0060648-Radiation Resulting in Increased Nuclear Fragmentation

To verify the ability of AZD6738 and KU-0060648 to abrogate radiation-induced DSB repair and prevent Rad51-mediated HR, we quantified the formation of γH2Ax and Rad51 foci in HCT116 p53+/+ and p53−/− cells.

**Figure 5:**
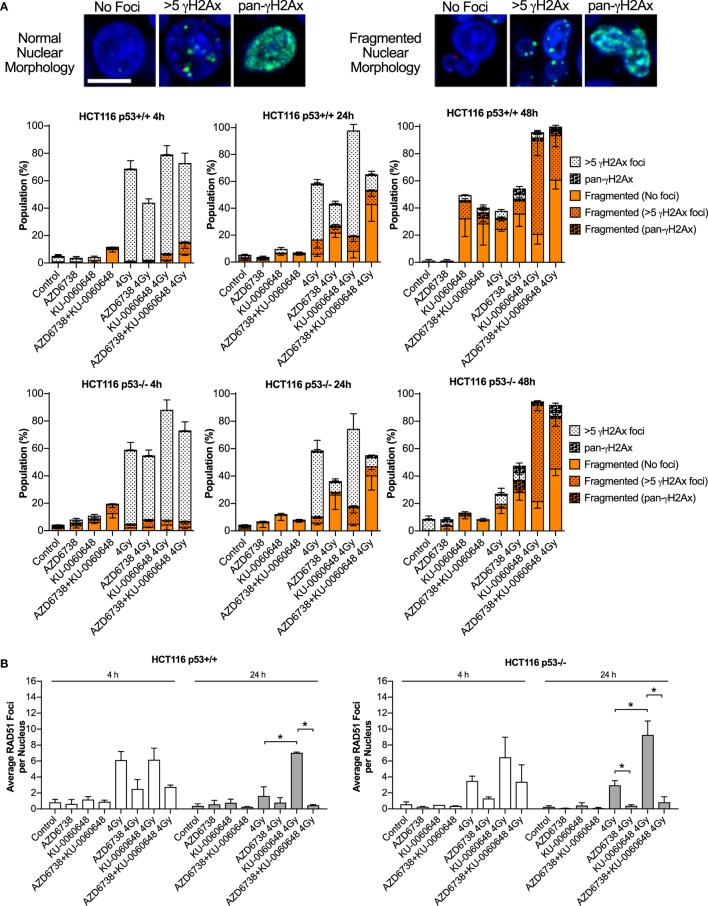

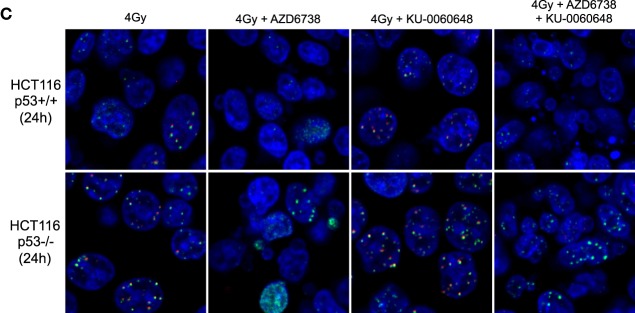
Modulation of H2Ax and RAD51 DNA repair focus formation by DNA-PK inhibition and ATR inhibition in combination with radiation. **(A)** Nuclear γH2Ax was imaged by confocal microscopy. Fixed samples were collected at the time points and treatments indicated in HCT116 p53+/+ and p53−/− isogenic cell lines. Nuclei were classified as having normal circular morphology or the presence of clear nuclear fragmentation such as micronuclei. A nucleus with multiple fragments was only scored as a single fragmented nucleus. Nuclei were further subclassified as having no γH2Ax, >5 γH2Ax foci, or having an unquantifiable level of γH2Ax staining referred to pan-γH2Ax positive staining. Example images of scoring are shown. Scale bar equals 10 µM with all images shown at the same magnification. DAPI used as nuclear counterstain. Data represent a minimum of three independent experiments ± SEM. Example images of scoring criteria are also shown. **(B)** RAD51 foci were imaged by confocal microscopy. RAD51 and nuclei were quantified by automated image quantification and expressed as the average RAD51 foci per nucleus. Data represent a minimum of two independent experiments at 4 h and three at 24 h ± SEM. Statistical analysis performed between indicated conditions by unpaired *t*-test *P < 0.05. **(C)** Larger example images of γH2Ax (green staining) and RAD51 (red staining), respectively, quantified in panels **(A,B)**.

Quantification of γH2Ax images classified nuclei as normal or abnormal in morphology with further sub-classification based on focal- or pan-γH2Ax staining (Figure 5A). Automated image quantification of γH2Ax was also performed at 4 and 24 h (Figure S2 in Supplementary Material). Data at 4 h were variable with no clear definitive changes apart from a trend for reduced radiation-induced γH2Ax due to the addition of AZD6738. At 24 h, there was some evidence that KU-0060648 may increase residual γH2Ax foci. At 24 h, AZD6738 reduced radiation-induced γH2Ax focus formation from both radiation alone and KU-0060648-radiation conditions. This was statistically significant in three out of four conditions where AZD6738 was added to radiation or radiation plus KU-0060648 (Figure S2 in Supplementary Material).

RAD51 images were analyzed at 4 and 24 h by automated image quantification. RAD51 focus formation is presented as the average number of foci per nucleus for each condition (Figure 5B). KU-0060648 at 24 h significantly increased levels of radiation-induced RAD51 foci in both cell lines. The addition of AZD6738 to radiation alone or KU-0060648-radiation strongly reduced Rad51 focus formation at both 4 and 24 h. The reduction in percentage of RAD51-positive cells observed at 4 h post-radiation is significantly greater than the reduction in S-phase possible over such a short time scale. However, at 24 h, the S-phase reduction as observed by BrdU staining for radiation and KU-0060648 (Figure 3A) indicates this may be a contributing factor to the drop in RAD51 under these conditions. This, however, was not the case for AZD6738 and radiation at 24 h as BrdU-positive cells maintained broadly similar levels to those of controls.

At 24 h, the addition of AZD6738 to radiation alone or KU-0060648-radiation manifests as an increase in nuclei classified as abnormal (Figure 5A). At 48 h, the total number of abnormal nuclei is similar between these two conditions, with the major difference being a substantial reduction in γH2Ax foci present due to the addition of AZD6738 to KU-0060648-radiation.

## Discussion

A number of DDR inhibitors have been extensively explored in preclinical studies. More recent DDR-targeting compounds have looked to exploit the high prevalence of a defective G1/S checkpoint in cancer cells compared with normal tissue. This has given rise to G2/M checkpoint-targeting agents such as ATR and CHK1 inhibitors. It has been hypothesized that maximal clinical efficacy of these agents is likely to be found in combination with exogenous DNA damage sources. Due to its position as a first-line treatment for HNSCC and many other cancers, radiation is a highly favorable source of this damage. A defective G1/S checkpoint through loss of function of p53 due to mutation or E6-mediated protein degradation by HPV infection is highly prevalent in HNSCC. We sought to better understand the role of p53 loss in determining radiosensitization by the combination of ATR and DNA-PK inhibitors.

Preclinical validation of both CHK1 inhibition (CHK1i) and ATR inhibition (ATRi) has shown them to be proficient radiosensitizers (8, 25–27). While ATRi and CHK1i have been shown to interfere with cell cycle arrest and HR, cancer cells may resort to alternative repair mechanisms such as NHEJ. While more error prone, this may present a mechanism capable of reducing the efficacy of ATRi in combination with radiation. For this reason, we sought to investigate if dual ATR and NHEJ inhibition, *via* DNA-PKi, was more effective in combination with radiation than ATRi alone.

The addition of ATRi to DNA-PKi-radiation or DNA-PKi to ATRi-radiation was shown to have the highest radiosensitization effect. This comparison includes the effect of radiosensitization with individual drugs combined with radiation. This is particularly compelling as both ATRi and DNA-PKi alone act as strong radiosensitizers, yet Bliss independence analysis suggests greater than expected cell kill was still possible with a second mechanistically independent DDR inhibitor.

Mechanistic data suggest the increased efficacy of dual ATRi and DNA-PKi in combination with radiation is based on both modulation to DNA repair focus formation and the resulting downstream nuclear fragmentation. DNA-PKi increased both RAD51 and γH2Ax focus formation after radiation. Interestingly, total γH2Ax phosphorylation decreased due to DNA-PKi in Western blots. This may indicate an increase in the number of unresolved DSBs and a reduction in the γH2Ax signal flanking the break site.

The appearance of large amounts of γH2Ax alongside caspase 3 or colocalization with TUNEL has been shown to be an apoptotic signal rather than DNA repair foci (28, 29). However, this does not appear to be the case as the addition of DNA-PKi reduces H2Ax by Western blot, though paradoxically clearly leads to increased γH2Ax foci (Figure 5). Foci and total γH2Ax appear to not be interchangeable measures of DNA DSBs. There may be more breaks but less extensive phosphorylation flanking the break site. Increased sub-G1 populations with the triple combination, despite no increase in cleaved caspase 3 or cleaved PARP, suggest a caspase-independent mechanism might be responsible for increased cell death. Mitotic catastrophe induced by paclitaxel in HCT116 was shown to increase sub-G1 populations in a caspase-independent manner (30). Loss of BUB1 leads to caspase-independent mitotic death due to treatment with the HSP90 inhibitor 17AAG (31). Due to the role of ATR as a cell cycle checkpoint, it is not unexpected that cell death may be, in part, driven by caspase-independent mitotic catastrophe.

The ability of ATR or CHK1i to abrogate G2 arrest and decrease RAD51 focus formation is well established. Mutations or DDR inhibitors that increase dependency on HR or G2 arrest are likely to combine favorably with ATR inhibition. This has proven to be the case with PARP inhibition (PARPi) which displays synergistic activity in combination with ATRi in BRCA-mutant ovarian (32, 33), SLFN11-negative prostate, leukemia, and Ewing’s sarcoma cell lines (34) as well as in HR-proficient cell lines (35). ATRi has also been shown to be synthetically lethal with ARID1A loss (36). ATRi in combination with PARPi leads to chromosome fragmentation as measured by chromatid breaks on metaphase spreads (32). ATRi in ARID1A−/− cells increases anaphase bridge formation and chromatid fragmentation on metaphase spreads (36). In this regard, our data on ATRi and DNA-PKi are phenotypically similar to that observed for both ARID1A loss and PARPi in combination with ATRi.

A sharp increase in nuclear fragmentation after aberrant mitotic transit appears to be a common finding when the ATR-CHK1 signaling pathway is disrupted in radiation combination studies (8, 37, 38). ATR plays a key role in the accumulation of ribonucleotide reductase subunit 2 and origin firing in S-phase. In early S-phase cells, ATRi can induce massive ssDNA accumulation, but later S-phase cells have been shown to trigger a DNA-PK- and CHK1-mediated backup pathway which suppresses origin firing (39). ATR has been shown to be critical for preventing nuclease cleavage of replication forks and severe nuclear fragmentation due to replication catastrophe (40–42). It is probable that dual inhibition of DNA-PK and ATR increases this phenomenon, manifesting as the increased nuclear fragmentation observed. Micronuclei generated as a result of nuclear fragmentation have been identified as displaying reduced replication and increased DNA damage during S-phase, as well as showing cell cycle dyssynchrony (8, 43). This is believed to be due to reduced nuclear import vs the primary nucleus or rupturing of the nuclear membrane (43, 44).

Previously published data have shown the ATR inhibitor AZD6738 is well tolerated at active doses *in vivo* in DNA-PK-deficient NOD scid gamma mice (8). Our data here show combined DNA-PKi and ATRi does not display obvious combined toxicity by clonogenic assay, with enhanced cell kill effects only observed due to the addition of radiation. This was most clearly illustrated by the ability of combining both drugs at 125 nM showing similar radiosensitization capability to each drug alone at the much higher dose of 500 nM. Taken together, these data may suggest a window for relative therapeutic benefit vs normal tissue outside the radiation field. G1/S checkpoint activation has been shown to occur slowly after radiation, in contrast to rapid activation of the G2/M checkpoint (45). Previous work in our lab on AZD6738 and radiation (8) has shown that S-phase or mitotic transition appears essential for radiosensitization activity. Tumor cells proficient in p53, such as HCT116, still readily enter and transit these phases of the cell cycle, and still do so for some time post-radiation. Non-proliferating normal tissue in G0/G1 represents a distinct p53-competent population not subject to the cell cycle-linked effects of radiation and ATRi in particular. Preclinical studies of AZD6738 in combination with olaparib *in vivo* have reported efficacy without weight loss compared with control groups (32). Of particular relevance to HNSCC will be any reports of worsening acute toxicities, such as oral mucositis, in preclinical and clinical studies since proliferative epithelial cells are likely to be the most sensitive normal cells within the radiation field to radiosensitizers. Careful assessment of normal tissue toxicities will be required for the entire class of radiosensitizers, especially in regard to normal, but proliferating, irradiated cells. In light of the similar phenotype observed, an open and recruiting clinical trial (NCT02264678) investigating the ATR and PARP inhibitors, AZD6738 and olaparib may help to inform any future studies into ATRi and DNA-PKi on both efficacy and any toxicity issues.

In summary, dual inhibition of DNA-PK and ATR represents a promising approach in combination with radiation. This combination appears to share mechanistic similarity with PARPi in combination with ATR inhibition. However, the evidence suggests dual ATRi and DNA-PKi driven radiosensitization is broadly similar irrespective of p53 status.

## Author Contributions

HH, MD, HB, JK, US, JP, HS, and MP were involved in experimental work. HH, MD, MM, and KJH were in involved in experimental planning discussions. HH, MM, and KJH were involved in manuscript preparation.

## Conflict of Interest Statement

The Institute of Cancer Research and Royal Marsden have received funding for a phase I study of AZD6738, which is partially funded by AstraZeneca.
