# Supplementary material for: Combined ATR and DNA-PK Inhibition Radiosensitizes Tumor Cells Independently of Their p53 Status
Source: Front Oncol. 2018 Jul 13;8:245. doi: 10.3389/fonc.2018.00245 (PMC6053502; doi:10.3389/fonc.2018.00245)
Supplement: Supplementary file 1 [file Image_1.PDF]

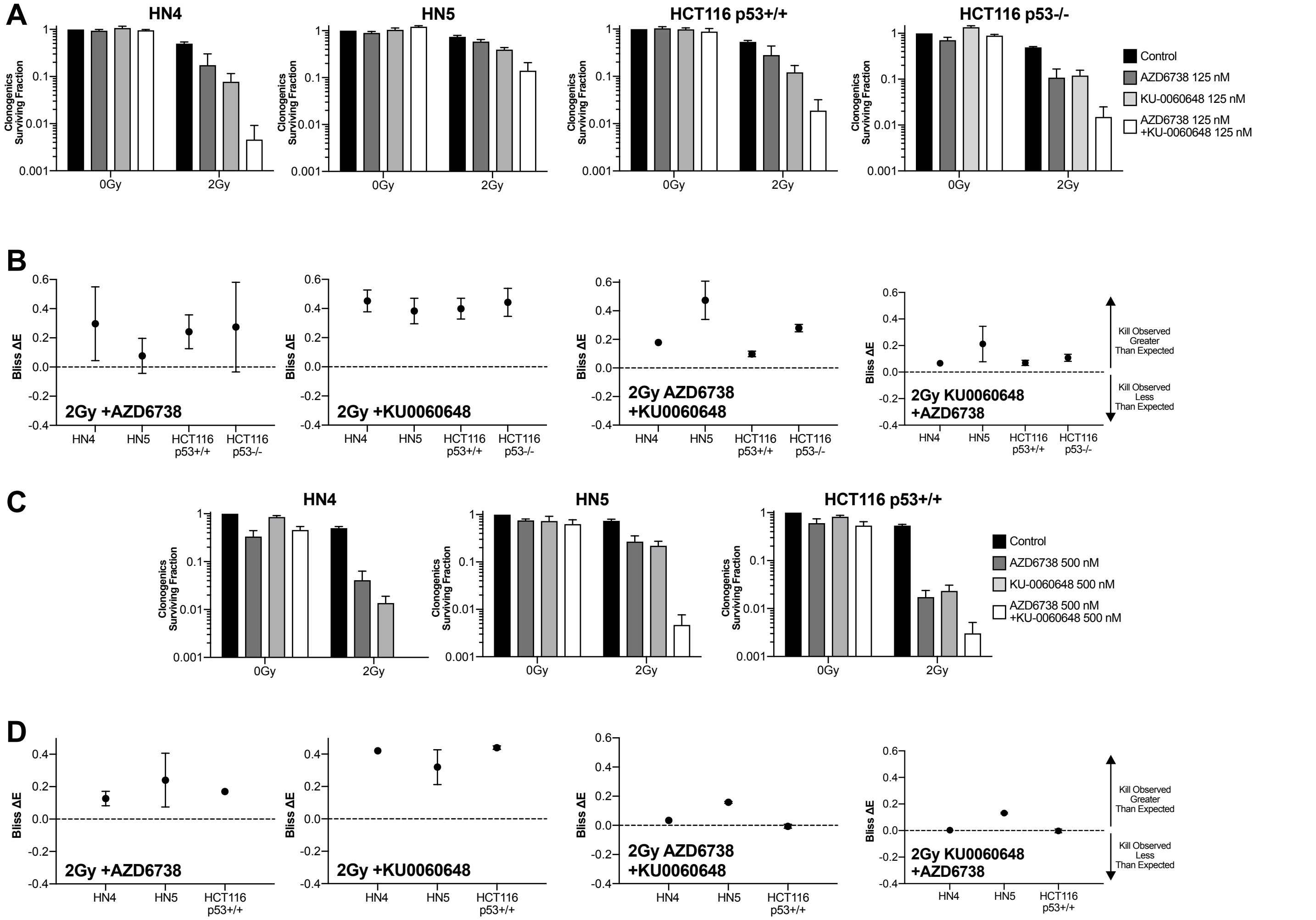

Figure S1. DNAPKi enhances radiosensitization when added to ATRi and radiation at an extended range of doses. (A, C) Clonogenic survival was determined for AZD6738, KU-0060648 and 2 Gy radiation, alone and in combination. AZD6738 and KU-0060648 were used at fixed doses 125 nM and 500 nM in panels A and C respectively. This is below and above the 250 nM used in the corresponding figure 1E. Values expressed as surviving fraction relative to untreated control. (B, D) Bliss analysis as described in figure 1. All panels represent a minimum of 3 independent biological repeats ± SEM, except Bliss analysis in B and D where error bars are ± 95% confidence intervals.
