# Supplementary material for: Combined ATR and DNA-PK Inhibition Radiosensitizes Tumor Cells Independently of Their p53 Status
Source: Front Oncol. 2018 Jul 13;8:245. doi: 10.3389/fonc.2018.00245 (PMC6053502; doi:10.3389/fonc.2018.00245)
Supplement: Supplementary file 2 [file Image_2.PDF]

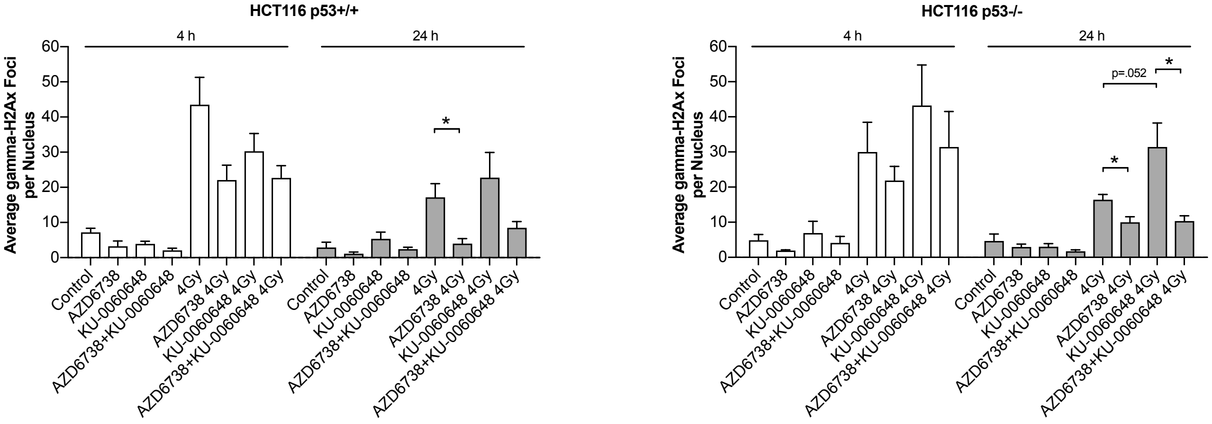

Figure S2. Modulation of γH2Ax DNA repair focus formation by DNAPKi and ATRi in combination with radiation. Nuclear γH2Ax foci were imaged by confocal microscopy. Fixed samples were collected at the time points and treatments indicated in HCT116 p53+/+ and p53-/- isogenic cell lines. 250 nM was used for both AZD6738 and KU-0060648, added 1 h before radiation. γH2Ax foci were quantified by automated image quantification and expressed as the average foci per nucleus with nuclear segmentation and counting based on DAPI staining. Data represent a minimum of 3 independent independent experiments ± SEM. Statistical analysis performed between indicated conditions by unpaired t-test * = P<0.05.
